# Seed Size‐Number Trade‐Off in Populations of a Cosmopolitan Species (*Oxybasis glauca*) From Xinjiang, China

**DOI:** 10.1002/ece3.73419

**Published:** 2026-04-07

**Authors:** Xiaolong Zhou, Ronghua Duan, Qiujin Ma, Fojun Zhang, Xiaohua Lv, Lijia Wang

**Affiliations:** ^1^ College of Ecology and Environment Xinjiang University Urumqi China; ^2^ Key Laboratory of Oasis Ecology Xinjiang University Urumqi China

**Keywords:** intraspecific variation, natural population, seed trait, trade‐off

## Abstract

The trade‐off between seed size and number is a fundamental reproductive strategy critical for species survival and distribution. While this trade‐off is well‐documented at the interspecific level, its manifestation within a single species (intraspecific scale) remains surprisingly scarce. In this study, we examined the interplay between seed size (mass) and seed number in 48 natural populations of the cosmopolitan species *Oxybasis glauca* along a broad environmental gradient in Xinjiang, northwestern China. We confirmed a significant negative correlation between seed size and seed number, reinforcing the classic trade‐off strategy in which individuals produce either few, large seeds or many, small seeds. Our results suggest that the negative correlation may reflect a fundamental resource‐partitioning trade‐off, where reproductive biomass allocation to seed size appears to occur at the direct expense of seed number. Notably, seed number exhibited extraordinary phenotypic plasticity, whereas seed size remained evolutionarily conserved, suggesting that fecundity is the primary trait for adaptive modulation. Furthermore, our results reveal that broad‐scale geographical, climatic, and soil gradients had negligible predictive power for reproductive variation. Instead, reproductive success was governed primarily by fine‐scale, human‐mediated resource availability within local microhabitats, with seed size, plant height and reproductive biomass acting as the primary functional links that translate local conditions into seed production. This synergistic strategy, pairing a stable seed size with a highly plastic seed number responsive to microhabitat conditions, underscores the remarkable capacity of *O. glauca* to thrive across diverse and disturbed environments, accounting for its success as a cosmopolitan species.

## Graphical abstract

This study provides strong intraspecific evidence for the classic seed size‐number trade‐off theory. Through an analysis of 48 natural populations of the cosmopolitan species *Oxybasis glauca* in Xinjiang, we explicitly confirmed its core strategy in reproductive investment: individuals produce either few, large seeds or many, small seeds, showing a significant negative correlation. The research further reveals that in adapting to diverse environments, the species' strategy tends to flexibly adjust seed number rather than alter seed size, which may be a key factor in its successful colonization on a global scale.

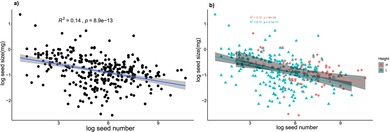

## 1 Introduction

Seeds represent a pivotal stage in the life history of flowering plants, encapsulating the genetic blueprint and nutrient reserves essential for the dispersal, establishment, and reproduction of the next generation, thereby ensuring population persistence (Silvertown and Dodd 1996; Nambara and Nonogaki 2012). Seed traits are central to plant reproductive strategies, as conceptualized in r‐K selection theory (Pianka 1970) and the leaf‐height‐seed (LHS) functional trait scheme (Westoby 1998). Among these traits, seed size and seed number are foundational, profoundly shaping reproductive strategies and overall plant fitness (Metz et al. 2010; Mironchenko and Kozlowski 2014). While seed size is directly linked to nutrient content and establishment success (Westoby et al. 1996; Moles et al. 2007), seed number determines dispersal efficacy and the probability of colonizing favorable sites (Schupp et al. 1989; Parciak 2002a).

A fundamental trade‐off governs these two traits. Plants face a resource allocation dilemma: producing larger, well‐provisioned seeds enhances seedling survival but limits the total number of offspring, whereas producing numerous smaller seeds maximizes dispersal but may compromise establishment (Venable and Lawlor 1980; Schupp et al. 1989; Parciak 2002a; Moles et al. 2007; Han et al. 2019). This resource‐based constraint forces species to evolve divergent strategies along a continuum from producing few, large seeds to many, small ones (Venable and Brown 1988, Venable 1992; Geritz 1995; Bufford and Hulme 2021; Bogdziewicz et al. 2023). Elucidating the patterns of this seed size‐number trade‐off is therefore crucial for understanding plant life‐history evolution, population dynamics, and community assembly processes (Leishman 2001; Moles et al. 2005; Venable and Rees 2009).

However, the manifestation of this trade‐off can be scale‐dependent, varying between interspecific (among species) and intraspecific (within species) levels (Germain and Gilbert 2014; Lázaro and Larrinaga 2018). While the trade‐off is well‐documented across diverse species (Smith and Fretwell 1974; Stearns 1976; Cadotte et al. 2006), its presence and strength at the population level are less certain, with some studies finding no significant trade‐off within species (e.g., Germain and Gilbert 2014). To date, research has predominantly focused on the interspecific scale, leaving the intraspecific dynamics comparatively unexplored. Studying this trade‐off at the population level is critical because it minimizes the confounding effects of deep phylogenetic history and divergent reproductive modes, allowing for a clearer assessment of how environmental factors and individual plasticity shape reproductive allocation.

Environmental conditions are known to be powerful modulators of the seed size‐number relationship (Ben‐Hur and Kadmon 2015; Gao et al. 2023). In resource‐limited habitats, plants often adopt a strategy of producing many small seeds to increase the odds of successful colonization (Metz et al. 2010). Conversely, in resource‐rich environments, investment shifts towards fewer, larger seeds to boost competitive ability (Dong et al. 2018). Abiotic stress gradients, such as increasing altitude or aridity, also impose constraints, typically leading to smaller seeds and accentuating the trade‐off (Pluess et al. 2005; Wang et al. 2021; Gao et al. 2023). Biotic factors, including reproductive mode (e.g., sexual vs. asexual) (Jakobsson and Eriksson 2000; Wang et al. 2024), genetic variation (Carta et al. 2016; Chen et al. 2018), and intraspecific variation (Cheplick 1995; Wang et al. 2022), further influence this dynamic (Jakobsson and Eriksson 2000; Dong et al. 2018). Given that seed size is often considered a relatively conserved trait (Sadras 2007), plants may primarily adjust seed number to adapt to fluctuating environments. Yet, this hypothesis requires rigorous empirical testing across broad environmental gradients.

To address these knowledge gaps, we investigated the seed size‐number trade‐off in the cosmopolitan annual herb *Oxybasis glauca* (L.) S. Fuentes et al. This species is globally successful, thriving in diverse and often harsh environments, including those with high salinity, drought, and thermal stress (Fuentes‐Bazan et al. 2012; Yao et al. 2019). By examining 339 individuals from 48 natural populations across a wide environmental gradient in Xinjiang, northwestern China, we sought to (1) determine whether a consistent seed size‐number trade‐off exists at the intraspecific level in *O. glauca* , and (2) disentangle the relative contributions of environmental factors and plant traits in shaping this reproductive strategy.

## 2 Materials and Methods

### 2.1 Study Area and Sampling Design

**FIGURE 1:**
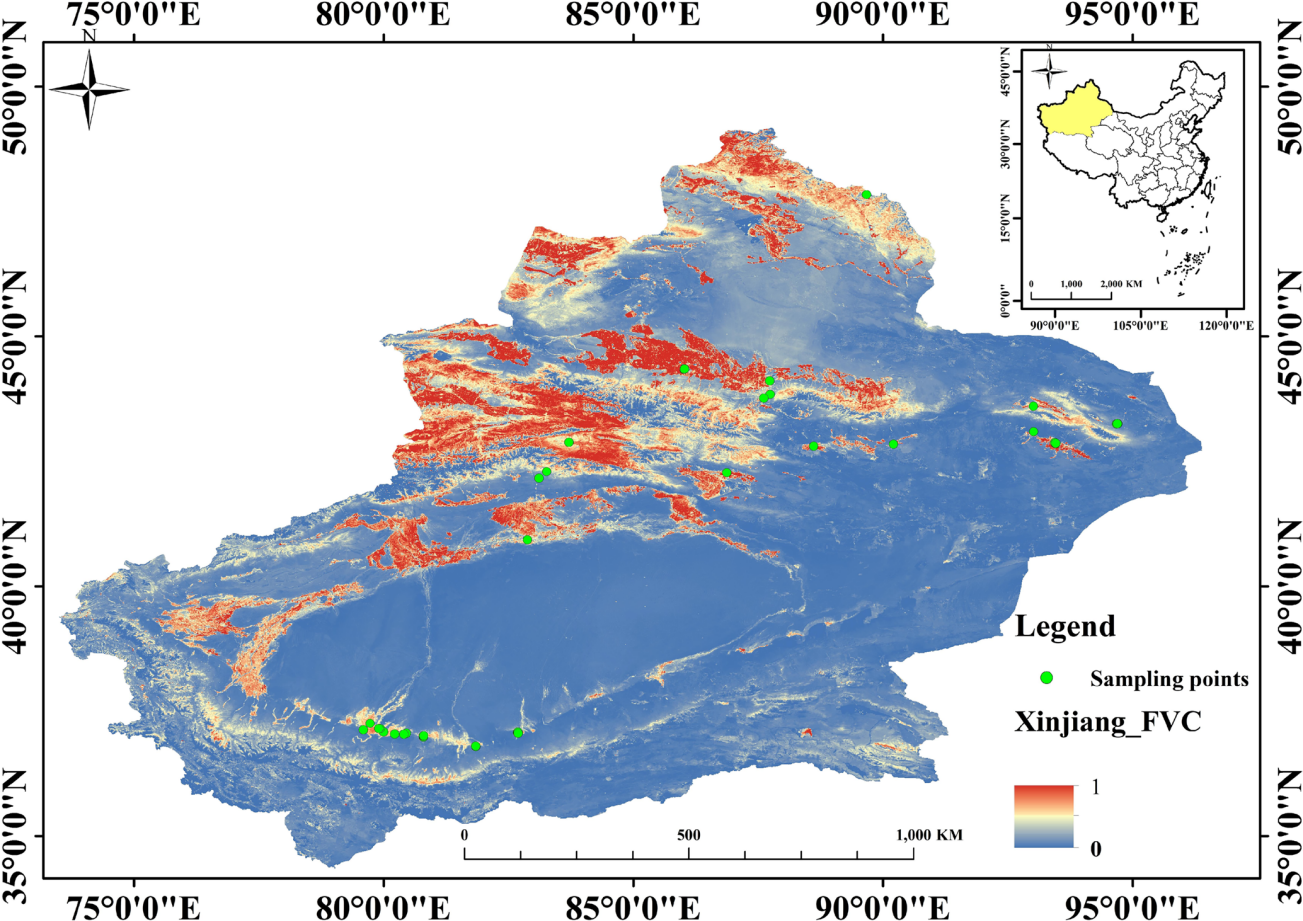
Geolocation of the *Oxybasis glauca* individuals sampled in Xinjiang, Northwestern China. FVC (Fractional Vegetation Cover) ranges from 0 to 1, representing the proportion of ground surface covered by vegetation, where 0 indicates bare soil and 1 indicates full vegetation coverage.

The study was conducted in the Xinjiang Uygur Autonomous Region, China. Field sampling occurred during the peak seed maturity period (late September to late October) in 2023 and 2024. We collected a total of 339 mature *O. glauca* individuals from 48 sites (Figure 1), spanning a wide geographical range (77.21°‐94.70° E, 36.79°‐48.87° N) and a significant elevational gradient (−51.7 m to 2408.2 m). The sites encompass a steep climatic gradient, with mean annual precipitation ranging from 32 to 307 mm and mean annual temperatures from −7.12°C to 15.21°C, based on a 10‐year climatic record (2014–2023). All sampling was conducted in publicly accessible areas where no permits were required.

### 2.2 Trait Measurements

In the field, we selected healthy, intact individuals with mature seeds. We measured plant height in situ and recorded the geographic coordinates (latitude, longitude, elevation) of each site using a GPS device. Habitat characteristics and disturbance levels were also noted. Based on the surrounding land‐use context and growing environment, habitats were classified into three types: agricultural margins, road margins, and urban green spaces. The entire reproductive portion of each plant was harvested, placed in an envelope, and transported to the laboratory.

In the laboratory, plant materials were air‐dried to constant weight. Reproductive biomass of each plant was measured to the 0.1 mg using an electronic balance. Seeds were then manually separated from the infructescence for each individual, and the total number of seeds (“seed number”) was counted. The total seed mass for each individual was weighed to the nearest 0.1 mg using an electronic balance. From these data, we calculated the average individual seed mass (hereafter “seed size”) by dividing the total seed mass by the total seed number.

### 2.3 Climate and Soil Data

Mean annual precipitation (MAP) and mean annual temperature (MAT) data for our study area were obtained from the National Earth System Science Data Center (https://www.geodata.cn/main). Specifically, climatic datasets with a spatial resolution of 1 km for the 2014–2023 period were retrieved (Peng et al. 2019). Based on the geographic coordinates of the 48 sampling sites, the MAP and MAT values were extracted using the Extract Values to Points tool within the Spatial Analyst module of ArcGIS 10.8. MAP is expressed in millimeters (mm) and MAT in degrees Celsius (°C).

Soil properties were extracted from the Harmonized World Soil Database (HWSD) v2.0, jointly developed by the Food and Agriculture Organization (FAO) and the International Institute for Applied Systems Analysis (IIASA) (Nachtergaele et al. 2008). For the 0–20 cm soil layer, data were processed using the Lookup Table function under the Raster Reclassification tool in the ArcGIS 3D Analyst module. To ensure spatial consistency at a 1 km resolution, the dominant soil type (the one with the highest area proportion) was identified and extracted for each site using the Extract Values to Points tool. Soil pH, soil total nitrogen (STN), and soil organic carbon (SOC) content were selected as key determinants to evaluate their influence on plant seed traits.

### 2.4 Statistical Analysis

All statistical analyses were performed in R version 4.2.2 (R Development Core Team 2019). Seed size, seed number and reproductive biomass were log‐transformed to meet assumptions of normality and homoscedasticity.

First, we used linear regression to quantify the overall relationship between seed size and seed number. To explore the potential influence of plant size, we categorized the dataset based on the mean plant height (“high” and “low” groups) and re‐assessed the trade‐off within each subgroup. We further employed linear regression models to evaluate how seed size and seed number were associated with reproductive biomass, as well as with climate and soil factors.

Second, we calculated the coefficient of variation (CV) for both seed size and seed number. We observed substantially higher variation in seed number than in seed size. Therefore, to identify the key drivers of seed number variation, we fitted Bayesian linear mixed‐effects models (LMMs) using the “brms” package (Bürkner 2017). In these models, seed number was the response variable. Biotic factors (log‐transformed seed size, plant height, reproductive biomass), geographical factors (longitude, latitude, altitude), climate factors (MAP, MAT), soil factors (pH, STN, SOC), and habitat types (agricultural margins, road margins, urban green spaces) were included as fixed effects, respectively. Sampling site was included as a random intercept to account for nonindependence among individuals from the same location. Models were run with default priors for 9999 MCMC iterations (4999 warm‐up) across four chains. We assessed the significance of fixed effects by examining their 95% credible intervals and calculated marginal and conditional *R* ^2^ to evaluate model fit.

To further explore the effects of biotic factors (seed size, height, reproductive biomass) on seed number variation in different local habitats (agricultural margins, road margins and urban green spaces), we performed multigroup structural equation modeling using “lavaan” package (Rosseel 2012). We constructed a path model where seed number was predicted by plant height, reproductive biomass, and seed size. To determine whether the structural relationships varied among habitat types, we compared a multigroup model with freely estimated paths against a constrained model where regression coefficients were fixed across all groups. Model fit was evaluated using the comparative fit index (CFI), Tucker–Lewis index (TLI), root mean square error of approximation (RMSEA), and standardized root mean square residual (SRMR). All parameters were estimated using the maximum likelihood (ML) method, and standardized coefficients were used to compare the relative importance of predictors across habitat types.

Data processing and visualization were performed using the “tidyverse” and “ggplot2” packages (Wickham 2009; Wickham et al. 2019), respectively.

## 3 Results

### 3.1 Seed Size‐Number Trade‐Off

**FIGURE 2:**
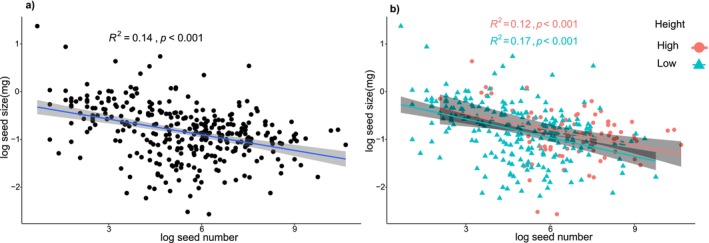
Relationships between seed size and seed number in *Oxybasis glauca* (a) population and (b) in high individual group and low individual group. The seed size and seed number data were log transformed. *R* ^2^ and *p* values are derived from linear regression.

**FIGURE 3:**
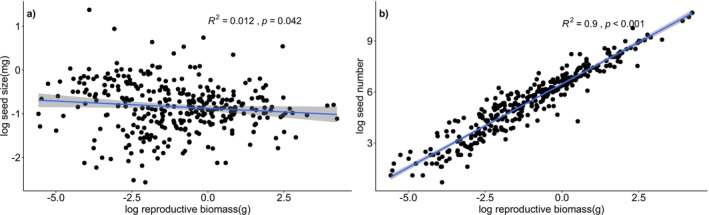
Relationships between (a) seed size and reproductive biomass and (b) seed number and reproductive biomass in *Oxybasis glauca* population. The seed size, seed number, and reproductive biomass data were log transformed. Black dots represent individual data points. *R* ^2^ and *p* values are derived from linear regression, panels with significant correlations (*p* < 0.05) include a blue regression line and a gray confidence interval.

**FIGURE 4:**
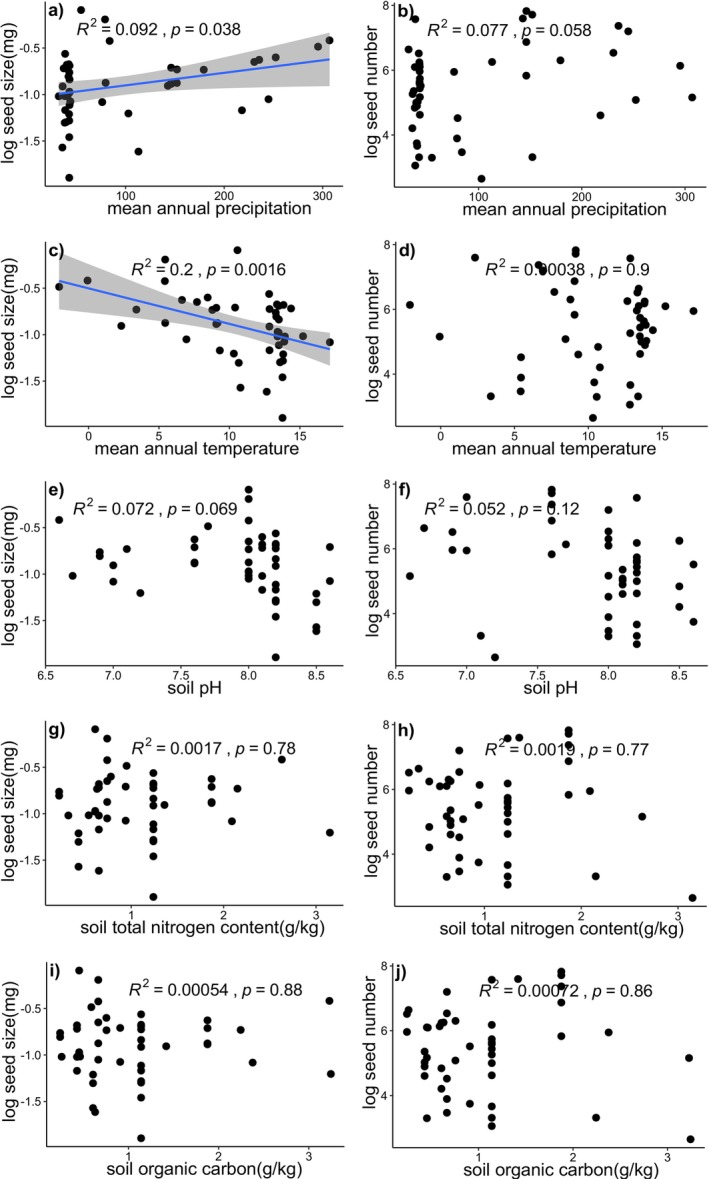
Relationships between seed size (a, c, e, g, i), seed number (b, d, f, h, j), and environmental factors at the site level, namely (a, b) mean annual precipitation, (c, d) mean annual temperature, (e, f) soil pH, (g, h) soil total nitrogen content, and (i, j) soil organic carbon. *R* ^2^ and *p* values are derived from linear regression, panels with significant correlations (*p* < 0.05) include a blue regression line and a gray confidence interval.

A negative correlation between seed size and seed number was observed across all sampled *O. glauca* individuals, with seed size being negatively correlated with seed number (*R* ^2^ = 0.14, *p* < 0.001; Figure 2a). This negative correlation persisted when individuals were stratified by plant height. The relationship was particularly pronounced in the low‐height subgroup, which showed a stronger correlation coefficient and a higher level of significance compared to the high‐height subgroup (Figure 2b). Moreover, seed size and seed number displayed divergent linear relationships with reproductive biomass—a weak negative correlation (*R* ^2^ = 0.012, *p* < 0.05) and a strong positive association (*R* ^2^ = 0.90, *p* < 0.001), respectively (Figure 3). While seed size was significantly associated with both MAP and MAT, seed number exhibited no significant association with either climatic or soil variables (Figure 4).

### 3.2 Higher Variability in Seed Number Than Seed Size

**FIGURE 5:**
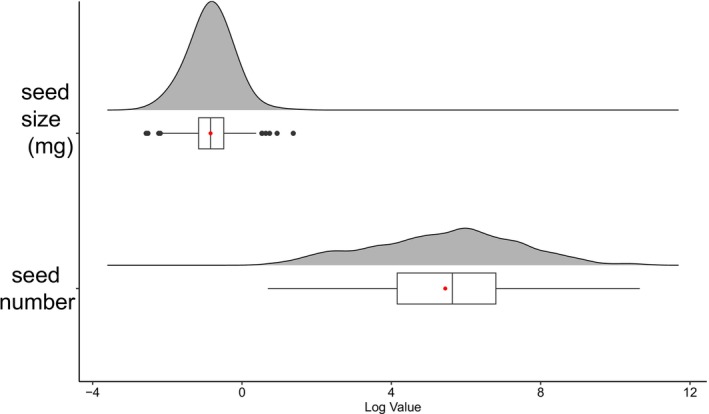
Data distribution of seed size and seed number. The seed size and seed number data were log transformed. The red points represent the mean value.

A key finding was the pronounced difference in variability between the two reproductive traits. Seed number was substantially more variable than seed size, as evidenced by a coefficient of variation (CV) more than twice as large (1.95 vs. 0.91) and a range several orders of magnitude wider (2–42,374 seeds vs. 0.08–3.95 units of mass) (Table 1, Figure 5). The lower skewness and kurtosis values for seed number further underscore its more symmetrical distribution compared with the highly skewed distribution of seed size. This pronounced plasticity in seed number suggests it is the primary trait through which *O. glauca* modulates its reproductive output in response to environmental conditions.

**TABLE 1:** Characteristics of data distribution for seed size and number of *Oxybasis glauca* population.

Statistical variable	Seed size (mg)	Seed number
Minimum	0.08	2
Maximum	3.95	42,374
Mean	0.51	1224
Median	0.43	280
Skewness	4.12	7.51
Kurtosis	29.99	68.21
Coefficient of variation	0.70	2.99

### 3.3 Drivers of Seed Number Variation

The Bayesian linear mixed‐effects model revealed that individual‐level biotic factors were the principal drivers of variation in seed number. Seed size, plant height and reproductive biomass emerged as significant predictors, collectively explaining 93.8% of the variance in seed number (Marginal *R* ^2^; Table 2). Similarly, habitat types explained 25.4% of the variation in seed number (Table S1). In contrast, large‐scale abiotic factors, including geographical coordinates (longitude, latitude, altitude) (Table S2), climatic variables (MAP, MAT) and edaphic properties (soil pH, STN, SOC) (Table S3), showed negligible explanatory power.

**TABLE 2:** Effects of seed size, plant height, and reproductive biomass on seed number variation.

Predictors	Estimates	CI (95%)
Seed size	−397.63	−674.35 to −153.13
Plant height	6.03	1.83–9.06
Reproductive biomass	550.58	532.67–569.61
**Random effects**		
*σ* ^2^	849369.86	
*τ*_00 site_	16584.98	
ICC	0.02	
*N*_site_	8	
Observations	339	
Marginal *R* ^2^/Conditional *R* ^2^	0.938/0.938	

*Note:* The results from the linear mixed models with the sampling sites as random variables based on the Bayes method. Model structure: Seed number ~ seed size + plant height + reproductive biomass + (1 | site).

Abbreviations: ICC, intraclass correlation coefficient; *N*_site_, number of sites.

## 4 Discussion

Our study offers three fundamental insights into the reproductive strategy of the cosmopolitan species *O. glauca* . First, our results reveal a robust negative correlation between seed size and seed number at the intraspecific level. This pattern arises primarily because the allocation of reproductive biomass to seed size reduces the potential investment in seed number (Figure 3). In contrast to a previous study (Knops et al. 2007), our results reveal that the negative correlation observed is not an artifact of environmental factors driving these two traits independently in opposite directions (Figure 4), but rather reflects a potential trade‐off in resource partitioning. Second, we demonstrate that seed number exhibits substantially greater phenotypic plasticity than seed size, suggesting it is the primary trait for adaptive modulation. Third, we reveal that this reproductive output is governed primarily by individual‐level biotic factors (seed size, plant height and reproductive biomass) (Table 2) rather than broad‐scale geographical factors (Table S2), climatic factors and soil physicochemical properties (Table S3). These findings collectively elucidate the mechanisms underpinning the ecological success of this globally widespread species.

### 4.1 Field Evidence for an Intraspecific Seed Size‐Number Trade‐Off Driven by Resource Limitation

The negative correlation between seed size and number is a cornerstone of plant life‐history theory (Venable 1992; Germain and Gilbert 2014; Lázaro and Larrinaga 2018), yet empirical evidence from within natural populations remain surprisingly scarce. Our study addresses this knowledge gap by providing empirical, field‐based evidence consistent with a potential seed size‐number trade‐off in *O. glauca* . We found that individuals consistently followed one of two strategies: producing many small seeds or few large ones. This pattern is consistent with the resource‐partitioning hypothesis, suggesting that the allocation of reproductive resources (i.e., reproductive biomass) to seed size may occur at the expense of seed number (Figure 3). Furthermore, our findings suggest that the observed negative correlation between seed size and number is not due to environmental factors influencing these traits independently in opposite directions (Figure 4). Instead, it appears to represent a potential trade‐off in how resources are allocated (Figure 3).

Two established theoretical frameworks have been proposed to explain the patterns of the seed size‐number trade‐off: the tolerance–fecundity trade‐off, which is based on stress tolerance (Muller‐Landau 2010; Lönnberg and Eriksson 2013), and the competition–colonization trade‐off, which is based on competitive ability (Geritz 1995; Rees and Westoby 1997). In our study, all sampling sites were located in arid regions, where low precipitation and high temperatures serve as the primary environmental stressors (Mittler 2006; Meslier and DiRuggiero 2019). However, our results demonstrated that seed size increased with MAP and decreased with MAT (Figure 4), which contradicts the predictions of the tolerance–fecundity trade‐off hypothesis. Consistent with the competition–colonization trade‐off, species or individuals with larger seeds typically exhibit higher germination and survival rates (Acevedo‐Limón et al. 2024); thereby bolstering their success during critical establishment phases (Parciak 2002b). We hypothesize that *O. glauca* individuals emerging from larger seeds possess superior competitive ability. Further research should focus on how these individuals secure such advantages and whether a higher frequency of dispersal events is essential for the successful establishment of smaller seeds.

Within the frameworks of both the tolerance‐fecundity and competition–colonization trade‐off hypotheses, resource limitation is generally considered a key prerequisite for a seed size‐number trade‐off, a theoretical expectation that is consistent with our empirical observations. The negative correlation between seed size and number was more evident in smaller individuals (low‐height group: *R* ^2^ = 0.17, high‐height group: *R* ^2^ = 0.12), suggesting that these plants may experience more stringent resource constraints. As an annual herb, all *O. glauca* individuals senesce in winter; therefore, the plant height recorded during our sampling period (late September to late October) serves as a reliable proxy to quantify the total resources acquired by each individual during the growing season. Moreover, the decisive role of resource limitation in governing trade‐off patterns was further evaluated by treating reproductive biomass as a finite resource pool (Leishman 2001; Sadras 2007), which individuals strategically partition between seed size and number (Venable 1992). In this study, seed size exhibited a positive linear relationship with reproductive biomass, whereas seed number showed a negative linear association, indicating that reproductive biomass acts as the limiting resource for individual seed production (Figure 3). As a pioneer species colonizing disturbed, often nutrient‐poor habitats, *O. glauca* faces intense resource limitation. Our results support the hypothesis that a constrained resource budget forces a stricter allocation choice, thereby strengthening the size‐number trade‐off (Venable and Brown 1988; Venable 1992; Kang et al. 2022). This resource‐dependent trade‐off is fundamental to understanding how individuals optimize fitness under varying environmental conditions (Ben‐Hur and Kadmon 2015; Zi et al. 2023).

### 4.2 High Intraspecific Variation in Seed Number Underlies Environmental Adaptation

A central finding of our study is the dramatically higher intraspecific variation in seed number compared to seed size. While seed size is an evolutionarily conserved trait closely linked to dispersal and establishment success (Moles et al. 2005; Sadras 2007), modifying it is risky and energetically costly. Instead, *O. glauca* appears to adapt to environmental heterogeneity primarily by adjusting its fecundity. The sheer range of seed production we observed, spanning four orders of magnitude (from 2 to > 40,000 seeds per plant), stands as a testament to this remarkable plasticity. This strategy allows the species to capitalize on favorable conditions with massive reproductive output while persisting under poor conditions with minimal investment (Silvertown 1989, Hodgson et al. 2020). We argue that this high plasticity in seed number, rather than seed size, serves as a primary mechanism facilitating the persistence and expansion of *O. glauca* across a vast range of global environments.

### 4.3 Local Resource Availability Outweighs Broad‐Scale Environmental Gradients in Shaping Seed Number

In contrast to previous studies (Wegrzyn et al. 2022; Zhao et al. 2022; Gao et al. 2023), we found that geographical factors (longitude, latitude, altitude) (Marginal *R* ^2^ = 0.022), climatic factors (MAT, MAP), and soil physicochemical properties (soil pH, STN, SOC) (Marginal *R* ^2^ = 0.092) had negligible predictive power for seed number variation (Tables S2, S3). Instead, individual plant height, seed size, and reproductive biomass were the dominant drivers, collectively explaining up to 93.8% of the variation in seed number (Table 2). In comparison, habitat types accounted for 25.4% of the variation in seed number (Table S1). We posit that this results from the specific ecological characteristics of *O. glauca* . The species thrives in human‐modified landscapes like agricultural margins, road margins and urban green spaces, where local conditions are dictated by anthropogenic activities. Specifically, in agricultural margins, intensive irrigation and fertilization largely alleviate local water and nutrient limitations. Under these conditions, *O. glauca* individuals attain greater plant height and higher reproductive biomass, which in turn promotes increased seed production (Figure S1). In contrast, individuals growing along road margins are typically constrained by simultaneous limitations in both water and nutrient availability, thereby modulating plant growth and reproductive output, with downstream consequences for seed production (Figure S1). In urban green spaces, although supplemental irrigation and fertilization are often present, *O. glauca* individuals are subjected to frequent mowing disturbances. Such recurrent biomass removal influences plant height and reproductive biomass accumulation, consequently affecting seed production (Figure S1). Overall, these intense, localized disturbances create a mosaic of microenvironments whose effects on resource availability likely override the more subtle signals of regional climate. Consequently, the reproductive success of an *O. glauca* individual is determined not by its geographical location or broad‐scale climatic and soil factors, but rather by its immediate access to key resources (e.g., water and nutrients) within its specific microhabitat. This pattern is consistently reflected in the robust associations observed among plant height, reproductive biomass, and seed production (Table S4).

## 5 Conclusions

This study provides empirical evidence consistent with a seed size‐number trade‐off at the intraspecific level in the cosmopolitan species *O. glauca* , suggesting that reproductive allocation may involve a balance between these two traits. We demonstrated that this species' core adaptive strategy relies not on the modification of its relatively stable seed size, but rather on the extraordinary phenotypic plasticity of its seed number. Our findings also demonstrate that in *O. glauca* , reproductive success is governed primarily by fine‐scale, human‐mediated resource availability within local microhabitats, rather than by broad‐scale geographical position or regional climatic and soil gradients, with plant height and reproductive biomass acting as the primary functional links that translate local conditions into seed production. This synergistic combination of a conserved seed size and a highly plastic seed number, responsive to specific microhabitat conditions, represents a powerful reproductive strategy for this species. It underscores the remarkable capacity of *O. glauca* to thrive across diverse and disturbed environments, accounting for its success as a cosmopolitan colonizer. Future research should focus on disentangling the specific micro‐environmental mechanisms and extending these intraspecific investigations to other widespread species.

## Author Contributions

**Xiaolong Zhou:** conceptualization (lead), data curation (equal), formal analysis (equal), funding acquisition (equal), writing – original draft (lead). **Ronghua Duan:** investigation (equal), methodology (equal). **Qiujin Ma:** formal analysis (equal), funding acquisition (equal), investigation (equal). **Fojun Zhang:** investigation (equal), methodology (equal). **Xiaohua Lv:** investigation (equal), methodology (equal). **Lijia Wang:** investigation (equal), methodology (equal).

## Funding

This work was supported by Cross‐regional Collaborative Innovation Program of Xinjiang Uygur Autonomous Region & Scientific Partnerships and International Collaboration Program of Shanghai Cooperation Organization, 2023E01010 and Natural Science Foundation of Xinjiang Uygur Autonomous Region, 2022D01C676, 2024D01C33.

## Ethics Statement

The authors have nothing to report.

## Conflicts of Interest

The authors declare no conflicts of interest.

## Supporting information


**Table S1:** Effects of habitat types (agricultural margins, road margins and urban green spaces) on seed number. The results from the linear mixed models with the sampling sites as random variables that based on the Bayes method. Model structure: seed number ~ agricultural margins + road margins + urban green spaces + (1 | site). ICC, intraclass correlation coefficient; *N*_site_, number of sites.
**Table S2:** Effects of longitude, latitude and altitude on seed number. The results from the linear mixed models with the sampling sites as random variables that based on the Bayes method. Model structure: seed number ~ longitude + latitude + altitude + (1 | site). ICC, intraclass correlation coefficient; *N*_site_, number of sites.
**Table S3:** Effects of climate and soil factors on seed number. The results from the linear mixed models with the sampling sites as random variables that based on the Bayes method. Model structure: seed number ~ mean annual precipitation + mean annual temperature + soil pH + soil total nitrogen content + soil organic carbon content + (1 | site). ICC, intraclass correlation coefficient, *N*_site_, number of sites.
**Table S4:** Structural equation model results quantifying the effects of height, reproductive biomass and seed size on seed number variation in three habitat types. The structural equation model constructed with R software using the “lavaan” package.
**Figure S1:** Means and standard errors of the effects of three habitat types on (a) seed number, (b) seed size, (c) height, and (d) reproductive biomass. Different letters indicate significant differences between different habitats (*p* < 0.05). AM: agricultural margins; RM, road margins; UGS, urban green spaces.

**Data S1:** ece373419‐sup‐0002‐DataS1.csv.

## Data Availability

All the required data are uploaded as Supporting Information.
