# Supplementary material for: Seed Size‐Number Trade‐Off in Populations of a Cosmopolitan Species (Oxybasis glauca) From Xinjiang, China
Source: Ecol Evol. 2026 Apr 7;16(4):e73419. doi: 10.1002/ece3.73419 (PMC13058232; doi:10.1002/ece3.73419)

**Supplementary Material**

Table S1 the effects of habitat types (agricultural margins, road margins and urban green spaces) on seed number. The results from the linear mixed models with the sampling sites as random variables that based on the Bayes method. Model structure: seed number ~ agricultural margins + road margins + urban green spaces + (1 | site). ICC: Intraclass Correlation Coefficient, N _site_: Number of sites.

| **Predictors** | **Estimates** | **CI (95%)** |
| --- | --- | --- |
| Agricultural margins | 8568.32 | 7076.48 - 9971.25 |
| Road margins | 405.52 | 405.52 - 1925.79 |
| Urban green spaces | 879.21 | 195.80 - 1670.24 |
| **Random Effects** | | |
| σ^2^ | 9508531.89 | |
| τ_00_ _site_ | 378798.81 | |
| ICC | 0.04 | |
| N _site_ | 8 | |
| Observations | 339 | |
| Marginal R^2^ / Conditional R^2^ | 0.254 / 0.295 | |

Table S2 the effects of longitude, latitude and altitude on seed number. The results from the linear mixed models with the sampling sites as random variables that based on the Bayes method. Model structure: seed number ~ longitude + latitude + altitude + (1 | site). ICC: Intraclass Correlation Coefficient, N _site_: Number of sites.

| **Predictors** | **Estimates** | **CI (95%)** |
| --- | --- | --- |
| Longitude | 2.04 | -23.67 - 28.61 |
| Latitude | 11.14 | -36.10 - 56.44 |
| Altitude | 0.89 | -0.13 - 1.95 |
| **Random Effects** | | |
| σ^2^ | 12057871.35 | |
| τ_00_ _site_ | 1561185.85 | |
| ICC | 0.11 | |
| N _site_ | 8 | |
| Observations | 339 | |
| Marginal R^2^ / Conditional R^2^ | 0.022 / 0.099 | |

Table S3 the effects of climate and soil factors on seed number. The results from the linear mixed models with the sampling sites as random variables that based on the Bayes method. Model structure: seed number ~ mean annual precipitation + mean annual temperature + soil pH + soil total nitrogen content + soil organic carbon content + (1 | site). ICC: Intraclass Correlation Coefficient, N _site_: Number of sites.

| **Predictors** | **Estimates** | **CI (95%)** |
| --- | --- | --- |
| Mean annual precipitation | 5.36 | -5.56 - 14.28 |
| Mean annual temperature | -99.41 | -320.28 - 74.63 |
| Soil pH | 143.38 | -221.95 - 652.43 |
| Soil total nitrogen content | 3288.33 | -1421.74 - 7275.36 |
| Soil organic carbon content | -2964.56 | -6490.44 - 1081.71 |
| **Random Effects** | | |
| σ^2^ | 12160347.81 | |
| τ_00_ _site_ | 379982.13 | |
| ICC | 0.03 | |
| N _site_ | 8 | |
| Observations | 339 | |
| Marginal R^2^ / Conditional R^2^ | 0.092 / 0.110 | |

Table S4 Structural equation model results quantifying the effects of height, reproductive biomass and seed size on seed number variation in three habitat types. The structural equation model constructed with R software using the 'lavaan' package.

P-value (χ^2^) = 0.812, GFI = 0.999, RMSEA < 0.001, SRMR < 0.011.

| **Habitat type** | **Predictor** | **Standard path coefficients** | **P value** |
| --- | --- | --- | --- |
| Agricultural margins | Height | -0.002 | 0.962 |
|  | Reproductive biomass | 0.984 | **<0.001** |
|  | Seed size | -0.075 | **0.018** |
| Road margins | Height | 0.105 | **0.001** |
|  | Reproductive biomass | 0.869 | **<0.001** |
|  | Seed size | -0.123 | **<0.001** |
| Urban green spaces | Height | -0.016 | 0.723 |
|  | Reproductive biomass | 0.805 | **<0.001** |
|  | Seed size | -0.130 | **<0.001** |

Figure S1. Means and standard errors of the effects of three habitat types on a) seed number, b) seed size, c) height and d) reproductive biomass. Different letters indicate significant differences between different habitats (P < 0.05). AM: agricultural margins; RM: road margins; UGS: urban green spaces.


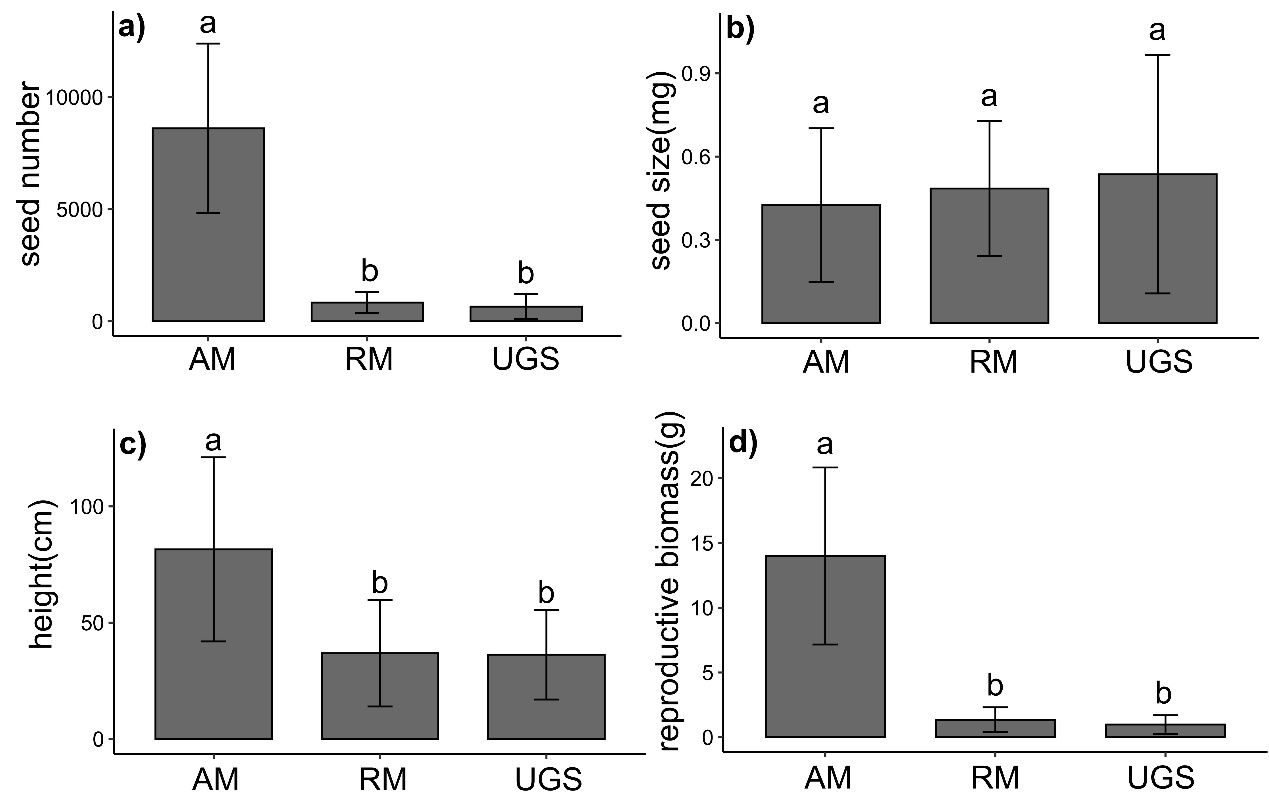

Supplement: Supplementary file 1 — Table S1: Effects of habitat types (agricultural margins, road margins and urban green spaces) on seed number. The results from the linear mixed models with the sampling sites as random variables that based on the Bayes method. Model structure: seed number ~ agricultural margins + road margins + urban green spaces + (1 | site). ICC, intraclass correlation coefficient; N site, number of sites. Table S2: Effects of longitude, latitude and altitude on seed number. The results from the linear mixed models with the sampling sites as random variables that based on the Bayes method. Model structure: seed number ~ longitude + latitude + altitude + (1 | site). ICC, intraclass correlation coefficient; N site, number of sites. Table S3: Effects of climate and soil factors on seed number. The results from the linear mixed models with the sampling sites as random variables that based on the Bayes method. Model structure: seed number ~ mean annual precipitation + mean annual temperature + soil pH + soil total nitrogen content + soil organic carbon content + (1 | site). ICC, intraclass correlation coefficient, N site, number of sites. Table S4: Structural equation model results quantifying the effects of height, reproductive biomass and seed size on seed number variation in three habitat types. The structural equation model constructed with R software using the “lavaan” package. Figure S1: Means and standard errors of the effects of three habitat types on (a) seed number, (b) seed size, (c) height, and (d) reproductive biomass. Different letters indicate significant differences between different habitats (p < 0.05). AM: agricultural margins; RM, road margins; UGS, urban green spaces. [file ECE3-16-e73419-s001.docx]
